# Longitudinal Analysis of Maternity Practices in Infant Nutrition and Care by Baby-Friendly Designation Status

**DOI:** 10.3390/children13101308

**Published:** 2026-09-26

**Authors:** Kathryn Wouk, Eileen FitzPatrick

**Affiliations:** 1 Pacific Institute for Research and Evaluation, Beltsville, MD 20705, USA; 2 Baby-Friendly USA, Albany, NY 12203, USA; efitzpatrick@babyfriendlyusa.org

**Keywords:** Baby-Friendly Hospital Initiative, breastfeeding support, maternity care practices, mPINC, lactation support, evidence-based care, health equity

## Abstract

**Highlights:**

**What are the main findings?**

- Hospitals that sustained Baby-Friendly designation had consistently higher mPINC survey scores than hospitals that were never designated or lost designation, and late adopters had the largest improvement in scores over time.
- Loss of Baby-Friendly designation was associated with significant decreases in evidence-based practice, as measured by the mPINC survey, including key breastfeeding-supportive domains of immediate postpartum care, feeding practices, and institutional management.

**What are the implications of the main findings?**

- Sustained Baby-Friendly designation may provide an important accountability structure for maintaining evidence-based maternity care practices, and the loss of designation is associated with lower levels of breastfeeding support.
- Public health and quality-improvement investments that help hospitals to maintain, obtain, or regain Baby-Friendly designation are needed to ensure access to critical breastfeeding support services and evidence-based care.

**Abstract:**

**Background/Objectives:** Evidence-based maternity care practices supportive of breastfeeding are critical for breastfeeding initiation, exclusivity, and continuation, and these practices are operationalized through the Baby-Friendly Hospital Initiative (BFHI) Ten Steps to Successful Breastfeeding. This study examined longitudinal trajectories of evidence-based maternity care practices supportive of breastfeeding by Baby-Friendly designation status among U.S. hospitals. **Methods:** Hospital-level data from the Centers for Disease Control and Prevention’s Maternity Practices in Infant Nutrition and Care (mPINC) surveys from 2018, 2020, 2022, and 2024 were analyzed. Hospitals completing at least two surveys were included in the analysis, categorized as having sustained designation, adopted designation late, lost designation, or never been designated. Longitudinal mixed-effects models estimated changes in total and domain-specific mPINC scores over time. **Results:** Among 2304 hospitals, 400 sustained Baby-Friendly designation, 190 adopted designation late, 160 lost designation, and 1554 were never designated. Hospitals with sustained designation maintained consistently high total mPINC scores from 2018 to 2024. Late adopters improved substantially, increasing 8.9 points (95% CI: 7.0,10.9), whereas hospitals that lost designation declined from 88.1 to 84.7, a change of −3.4 points (95% CI: −5.6, −1.3). Declines among hospitals that lost designation were most evident in immediate postpartum care, feeding practices, and institutional management domains. Never-designated hospitals’ scores improved modestly but remained below those of the sustained Baby-Friendly hospitals across survey years and domains. **Conclusions:** Sustained Baby-Friendly designation was associated with stable, high implementation of breastfeeding-supportive maternity practices, whereas loss of designation was associated with measurable declines in key breastfeeding-supportive domains. Maintaining or regaining designation may protect access to critical breastfeeding support services across communities.

## 1. Introduction

Breastfeeding is the normative pattern of infant feeding, supporting infant growth, development, and maternal postpartum recovery and conferring well-established maternal and infant health benefits: breastfeeding provides optimal infant nutrition that is associated with reduced risks of respiratory infections, otitis media, gastrointestinal illness, type 1 diabetes, and sudden infant death syndrome, while also reducing maternal risks of breast and ovarian cancers, type 2 diabetes, and hypertension [1,2,3]. Major medical and public health organizations in the United States (US) recommend exclusive breastfeeding for six months with continued breastfeeding for two years or beyond as long as mutually desired by mother and child [4,5,6,7,8]. The maternity care practices of immediate, uninterrupted skin-to-skin contact, early initiation of breastfeeding, avoidance of non-medically indicated supplementation, and 24 h rooming-in have been shown to enable higher rates of breastfeeding initiation, exclusivity, and duration [9,10,11].

These practices are operationalized in the Baby-Friendly Hospital Initiative (BFHI) Ten Steps to Successful Breastfeeding. The Ten Steps include policies and clinical practices that protect, promote, and support breastfeeding across the prenatal, intrapartum, and immediate postpartum period [12,13]. In the US, Baby-Friendly USA, a national non-profit organization, serves as the accrediting body for the BFHI. Baby-Friendly designation via BFUSA is a key mechanism for advancing evidence-based maternity care practices aligned with the Ten Steps, and adherence to these practices is associated with higher exclusive breastfeeding rates and improved maternal–infant health outcomes [14,15,16]. Yet maintaining designation as a Baby-Friendly hospital requires time and resources, as hospitals must undergo reassessment every five years, and staff turnover and resource constraints contribute to uneven implementation and occasional loss of designation [17,18]. National surveillance indicators suggest that Baby-Friendly hospital designation has decreased from a level representing 29% of U.S. births in 2021 [19] to 22% in 2025 [12]. Re-designation is a critical accountability and sustainability checkpoint, and loss of designation may signal erosion of evidence-based maternity care practices that support optimal breastfeeding.

Since 2007, the Centers for Disease Control and Prevention (CDC) has tracked maternity practices and policies that support breastfeeding through the Maternity Practices in Infant Nutrition and Care (mPINC) survey. Administered approximately every two years, mPINC assesses evidence-based maternity care practices consistent with the Ten Steps such as immediate postpartum care, rooming-in, feeding education and support, and institutional policies [20]. Multiple national analyses have demonstrated a relationship between higher mPINC scores and exclusive breastfeeding rates across survey cycles [21,22,23]. Despite this evidence linking maternity practices to improved breastfeeding outcomes, it remains unknown whether hospitals that lose Baby-Friendly designation subsequently experience declines in maternity practices supportive of breastfeeding compared with hospitals that sustain designation over time.

The objective of this study was to assess whether maternity care practices supportive of breastfeeding differed longitudinally among hospitals with different Baby-Friendly designation status. We used hospital-level data from four mPINC survey cycles to examine changes in overall and domain-specific scores over time by Baby-Friendly designation status.

## 2. Materials and Methods

### 2.1. Data Source and Study Sample

For this analysis, we used hospital-level data from the CDC mPINC surveys administered in 2018, 2020, 2022, and 2024. For each survey cycle, the CDC contacts hospitals and asks the mother–baby nurse manager or labor and delivery manager to identify the staff member most knowledgeable about infant feeding practices and maternity care policies to complete the survey [24]. Across these four cycles, mPINC surveys from 2746 unique hospitals were available with 2045 hospitals participating in 2018, 2103 in 2020, 1994 in 2022, and 2070 in 2024 (and response rates of 70%, 75%, 72%, and 78%, respectively [25]. To evaluate longitudinal changes in maternity care practices and Baby-Friendly designation status, we restricted the analytic sample to the 2370 hospitals from across the U.S. that completed at least two mPINC surveys between 2018 and 2024. This study was determined to be exempt from review by the Institutional Review Board at the Pacific Institute for Research and Evaluation (IRB #2314171-1).

### 2.2. Measurement

The survey includes six core domains that assess hospital maternity care practices associated with breastfeeding outcomes. These domains include (1) Immediate postpartum care; (2) Rooming-in; (3) Feeding practices; (4) Feeding education and support; (5) Discharge support; and (6) Institutional management (comprising staff training, breastfeeding data collection, formula acquisition, and policies related to breastfeeding) [20]. The total mPINC score indicates a hospital’s overall alignment with evidence-based maternity care practices that support optimal infant feeding, and it is the average of the six domain scores. Scores range from 0 to 100, with higher scores indicating greater alignment with supportive practices.

In this analysis, the primary outcome was the hospital’s total mPINC score for each survey year, and it was used to examine differences in maternity care practices by hospital Baby-Friendly USA designation status. Domain-specific scores were also analyzed to characterize which areas of maternity care practice contributed to observed differences in the total score and to inform potential targets for quality improvement. Domain-specific scores were not calculated if half or more of the items within that domain were did not have a score. The total mPINC score was not calculated if any domain score was missing, consistent with CDC scoring guidance.

Baby-Friendly designation status was determined using mPINC item A3 for each survey year: “Is your hospital currently designated as ‘Baby-Friendly’ by the Baby-Friendly Hospital Initiative (BFHI)?”. For each hospital, designation status was assessed across all completed survey years to characterize longitudinal designation trajectories. Hospitals were categorized into four mutually exclusive longitudinal designation cohorts based on monotonic designation patterns across completed survey years: (1) Never designated: not designated in any completed survey year; (2) Sustained designation: designated in all completed survey years; (3) Lost designation: transitioned from designated to not designated without subsequently regaining designation; and (4) Adopted late: transitioned from not designated to designated without subsequently losing designation. Hospitals with non-monotonic designation trajectories were excluded from cohort analyses (N = 66). Cohort assignment was based solely on the survey waves each hospital completed; designation status was not imputed for non-completed waves.

Hospital characteristics, including hospital ownership type, teaching hospital status, highest level of neonatal care, geographic region, and hospital size (via annual live births) were used for descriptive and adjusted analyses. Characteristics reflect the most recent available mPINC survey response for each hospital.

### 2.3. Statistical Analyses

We built a longitudinal mixed-effects model to examine change in total and domain-specific mean mPINC scores over time by Baby-Friendly designation cohort. We compared a linear time specification with a categorical time specification using the Akaike Information Criterion (AIC). The categorical-time model demonstrated substantially better fit, indicating non-linear change in scores across years. Therefore, time was represented using indicator variables for survey year (2018, 2020, 2022, and 2024), allowing for flexible, non-linear changes in mPINC scores over time.

Models included a hospital-level random intercept to account for clustering of repeated measures within hospitals and an autoregressive correlation structure to account for within-hospital temporal correlation. Using categorical time models, we estimated predicted mean total and domain-specific mPINC scores for each cohort–year combination, and contrasts were used to estimate within-cohort change from 2018 to 2024 as well as between-cohort differences at each time point. Statistical significance was evaluated using two-sided tests with α = 0.05. All differences were expressed as the difference between 2024 and 2018 to preserve temporal interpretation and ensure consistent directionality of change.

To assess potential confounding, we sequentially adjusted the primary mixed-effects model by each of the hospital characteristics described above using the most recent survey response available for each hospital. Across all adjusted models, effect estimates for Baby-Friendly designation cohort and survey year differed by less than 5% from the base model after adjustment, with no changes in direction or statistical significance. Therefore, hospital characteristics were not retained in the final models to preserve parsimony.

## 3. Results

A total of 2304 maternity hospitals were included in the analysis, including 400 hospitals that sustained Baby-Friendly designation, 190 that adopted designation late, 160 that lost designation, and 1554 that were never designated (Table 1). Hospitals that sustained designation were more likely to be government or military institutions and teaching hospitals compared with facilities that were never Baby-Friendly. Hospitals that sustained designation also tended to have higher levels of neonatal care and larger delivery volumes. In contrast, hospitals that were never designated were more commonly smaller-volume, for-profit, private hospitals and more frequently reported lower levels of neonatal care. Regional distribution varied modestly across cohorts, with sustained designation hospitals somewhat more concentrated in the Northeast and Western regions compared with hospitals that were never designated (Table 1).

Predicted total mPINC scores changed significantly over time within most hospital cohorts except for the sustained cohort, where they remained basically stable over time. Between 2018 and 2024, “Adopted late” hospitals increased by 8.9 points (95% CI: 7.0, 10.9), hospitals that lost designation declined by 3.4 points (95% CI: −5.6, −1.3), and “No designation” hospitals increased by 5.5 points (95% CI: 4.8, 6.2) (Table 2).

Across the 2018–2024 surveys, predicted scores were consistently highest among hospitals with sustained Baby-Friendly designation, followed by those that adopted designation later, those that lost designation, and finally hospitals that were never designated. Total mPINC scores remained stable and high among sustained Baby-Friendly hospitals, increased over time among late adopters, and remained lower but increasing over time among never-designated hospitals, while scores among hospitals that lost designation declined modestly, diverging from the background trajectory (Figure 1).

Domain-specific patterns were similar, with the largest improvements observed among late adopters in institutional management and rooming-in, and persistently lower scores among never-designated hospitals across all domains. Differences between cohorts were most pronounced in institutional management, discharge support, and rooming in, whereas gaps were narrower for immediate postpartum care, feeding education and support, and feeding practices (Figure 2).

## 4. Discussion

In this longitudinal analysis of U.S. hospitals participating in multiple mPINC survey cycles from 2018 through 2024, hospitals that sustained Baby-Friendly designation maintained consistently high levels of evidence-based maternity care practices supportive of breastfeeding, while hospitals that lost designation experienced significant declines overall and in key domains central to breastfeeding support, including immediate postpartum care, feeding practices, and institutional management. These findings demonstrate longitudinal associations between Baby-Friendly designation status and maternity care practices as measured through the mPINC survey. Out of a 100-point total mPINC score, hospitals that sustained Baby-Friendly designation maintained total scores above 90 across the study period (91.5–92.5), while hospitals that adopted designation later demonstrated substantial improvements over time, with scores increasing from 78.4 to 87.3. In contrast, hospitals that lost designation had significant declines in total mPINC scores, and hospitals that were never designated had mPINC scores that remained about 14 points below those of hospitals with sustained designation. Given that a 10-point increase in a modified mPINC score is associated with a 4.4 percentage point increase in exclusive breastfeeding [21], these sizable mPINC score differences by Baby-Friendly designation status highlight the potential importance of structured public health programs that improve maternal and infant health outcomes.

The practices captured by mPINC are closely aligned with the Ten Steps and with the clinical processes through which hospitals support breastfeeding initiation, exclusivity, and continuation [21,26]. The decline for hospitals that lost designation was evident not only in the total score, but also in domains central to both bedside care and the organizational infrastructure that sustains it. Declines in immediate postpartum care and feeding practices are particularly consequential given the established links between skin-to-skin contact, avoidance of non-medically indicated supplementation, and breastfeeding outcomes [27,28,29]. Beyond breastfeeding outcomes, reduced implementation of these practices may also have consequences for maternal caregiving capacity, particularly among the most vulnerable mothers [30]. The parallel decline in institutional management, reflecting staff training, breastfeeding data monitoring, and policy infrastructure (see Section 2.2), suggests that loss of designation may weaken bedside practice and the administrative systems that sustain them over time. With most mothers in the U.S. initiating breastfeeding (86.1% for infants born in 2023 [19], maintaining this infrastructure across all hospitals may help families reach their infant feeding goals.

The pattern among late-adopting hospitals provides additional evidence that adoption of Baby-Friendly designation is associated with meaningful improvements in measured practices. Late adopters began the study period with lower total and domain-specific scores than sustained Baby-Friendly hospitals but showed large gains by 2024, particularly in institutional management and rooming-in. These domains reflect hospital-level policies, staff responsibilities, and care routines that often require system-level change rather than isolated clinical education. Their improvement among late adopters is consistent with the interpretation that Baby-Friendly implementation may contribute to organizational change when hospitals make active investments in policy, training, monitoring, and workflow redesign. At the same time, late adopters did not reach the consistently high performance observed among hospitals that sustained designation throughout the study period, reinforcing the potential importance of continued monitoring and reassessment after initial gains are achieved.

Findings from this study are consistent with prior research showing that Baby-Friendly implementation is associated with more breastfeeding-supportive hospital care. Two recent systematic review and meta-analyses found that Baby-Friendly implementation was associated with improved early initiation of breastfeeding and exclusive breastfeeding at later time points, although effect sizes varied by context and implementation fidelity [31,32]. Alongside our findings, this literature suggests that the benefits of Baby-Friendly designation may depend not only on initial implementation, but also on sustained adherence, staff competency, and institutional reinforcement over time.

Differences in hospital characteristics across designation cohorts also have implications for access to breastfeeding supportive care. Patterson et al. found that Baby-Friendly designation is associated with increases in exclusive breastfeeding in hospitals located across all neighborhood socioeconomic levels [16]. This finding is particularly consequential because breastfeeding rates are lower among socioeconomically disadvantaged women, leaving their infants disproportionately exposed to the health risks associated with reduced or absent breastfeeding [33]. Targeted expansion of Baby-Friendly designation may help narrow this exposure gap and reduce the disproportionate burden of preventable infant morbidity among disadvantaged communities [34]. In our study, hospitals sustaining Baby-Friendly designation tended to be larger, higher-acuity, and more often teaching institutions, whereas hospitals that were never designated were more commonly smaller-volume and for-profit hospitals with lower levels of neonatal care. Although adjustment for measured hospital characteristics did not materially change our estimates, these descriptive differences suggest that the capacity to achieve and maintain Baby-Friendly designation may be unevenly distributed across the maternity care system [35]. If smaller-volume hospitals or those operating with fewer resources are less likely to sustain evidence-based practices supportive of breastfeeding, women giving birth in those settings may have less reliable access to breastfeeding-supportive care. Sustained investment in technical assistance, staff training, and quality-improvement infrastructure may be necessary to ensure that the benefits of Baby-Friendly-aligned maternity care are available across diverse hospital settings.

### 4.1. Limitations

The findings should be interpreted within the context of several limitations. First, mPINC data are self-reported by hospital staff and may be subject to reporting bias. Second, Baby-Friendly designation status was measured using the mPINC survey item rather than independently verified designation records for each year, which could introduce misclassification. Third, the designation cohorts were defined using completed survey waves; hospitals with non-monotonic designation trajectories were excluded from cohort analyses, and survey nonresponse may have influenced observed patterns. Fourth, our study evaluated hospital practices and policies rather than patient-level breastfeeding outcomes, so the analysis cannot directly estimate the effect of designation loss on breastfeeding initiation, exclusivity, or duration. Finally, although measured hospital characteristics did not meaningfully confound the main estimates, unmeasured factors such as leadership commitment, staffing models, lactation consultant availability, local breastfeeding coalitions, and state policy environments may have influenced both designation trajectories and mPINC scores.

Cohort classification relied on observed designation status at completed survey waves only; for hospitals that did not complete all four surveys, designation status during intervening, non-completed waves is unknown. It is possible that some hospitals classified as Sustained or Never Designated experienced an undetected change in status during an unobserved wave, which could not be captured by our monotonic classification rule, though when we re-estimated the primary model restricted to the hospitals that completed all four survey waves, there were no significant changes in point estimates and 95% confidence intervals, suggesting our findings are unlikely to be explained by incomplete survey participation. For hospitals classified as Adopted Late or Lost Designation, the observed transition in designation status could have occurred at any point within the interval between the two completed survey waves that bracketed it. While this interval was not always a single, consecutive survey cycle, the observed transition did occur in an adjacent two-year survey window for 94% of the Adopted Late hospitals and 86% of the Lost Designation hospitals. This imprecision in the timing of transition is additional limitation of the monotonic classification approach.

### 4.2. Strengths

This study has also several strengths. We used national hospital-level surveillance data, although the survey is a long-standing national surveillance system collected across four mPINC survey cycles, allowing examination of longitudinal trajectories rather than cross-sectional differences alone. The analytic approach distinguishes hospitals that sustained, adopted, lost, or never achieved designation, providing a more nuanced assessment of Baby-Friendly status than a simple designated versus non-designated comparison. The inclusion of domain-specific scores also helps identify which areas of practice changed most over time and where hospitals may need targeted support. Finally, the use of mixed-effects models accounts for repeated measures within hospitals and allows flexible modeling of non-linear change across survey years.

### 4.3. Implications

These findings have important implications for public health practice and policy. Sustained Baby-Friendly designation was consistently shown to correspond to high levels of maternity care practices supportive of breastfeeding over time. Loss of Baby-Friendly designation is associated with decreased compliance with recommended maternity practices to support breastfeeding. Re-designation processes, ongoing monitoring, and the external accountability may prevent erosion of practices after initial implementation. At the same time, the lower scores among never-designated hospitals indicate that broader dissemination of Baby-Friendly-aligned practices is critical and consistent with recommendations from the National Academy of Sciences that all U.S. maternity care facilities implement the Ten Steps as the standard of care to meet national breastfeeding objectives and improve health equity [36].

These findings also highlight the need for implementation research focused on why some hospitals are unable to maintain and sustain Baby-Friendly designation. Prior work has noted that loss of Baby-Friendly designation occurs [16] and that additional research is needed to understand the organizational, financial, staffing, and leadership factors that contribute to unsuccessful maintenance [17,37]. Potential mechanisms include staff turnover, reduced leadership engagement, lack of prioritization of staff breastfeeding training and competency, and insufficient internal monitoring after the initial designation period. Future research should examine the reasons hospitals lose designation, identify supports that promote successful re-designation, and link hospital-level designation trajectories to patient-level breastfeeding and equity outcomes.

## 5. Conclusions

Findings from this longitudinal analysis of national data indicate that loss of Baby-Friendly designation may reduce access to critical breastfeeding support services for women across the communities those hospitals serve. Sustained designation, re-designation, and ongoing quality-improvement support may therefore be important mechanisms for protecting community access to breastfeeding-supportive maternity care. Public health investments that help hospitals maintain or regain Baby-Friendly designation are especially important for ensuring that women and infants across diverse geographic, socioeconomic, and hospital settings have access to evidence-based breastfeeding support.

## Figures and Tables

**Figure 1:**
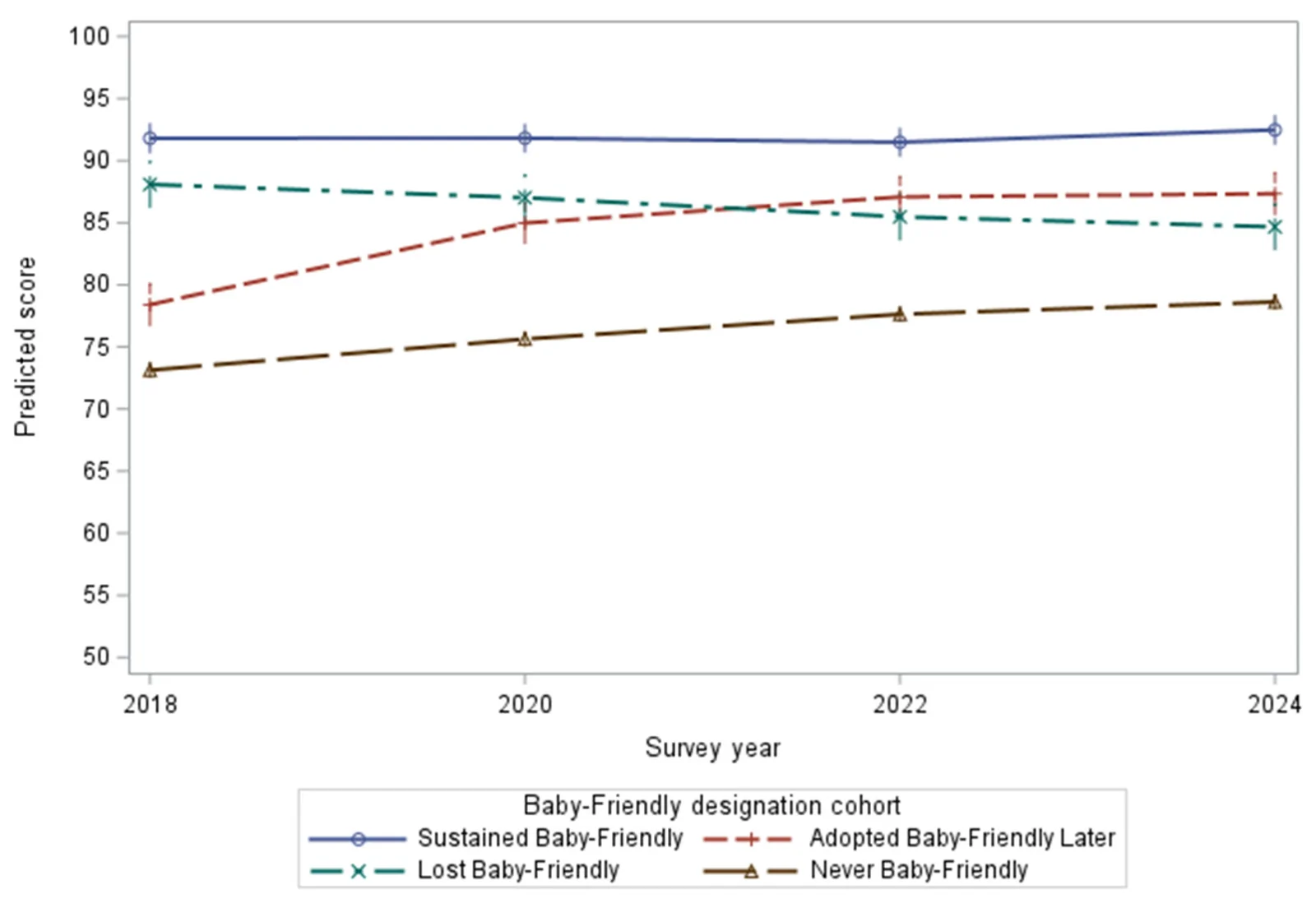
Total mPINC score by Baby-Friendly designation cohort, 2018–2024.

**Figure 2:**
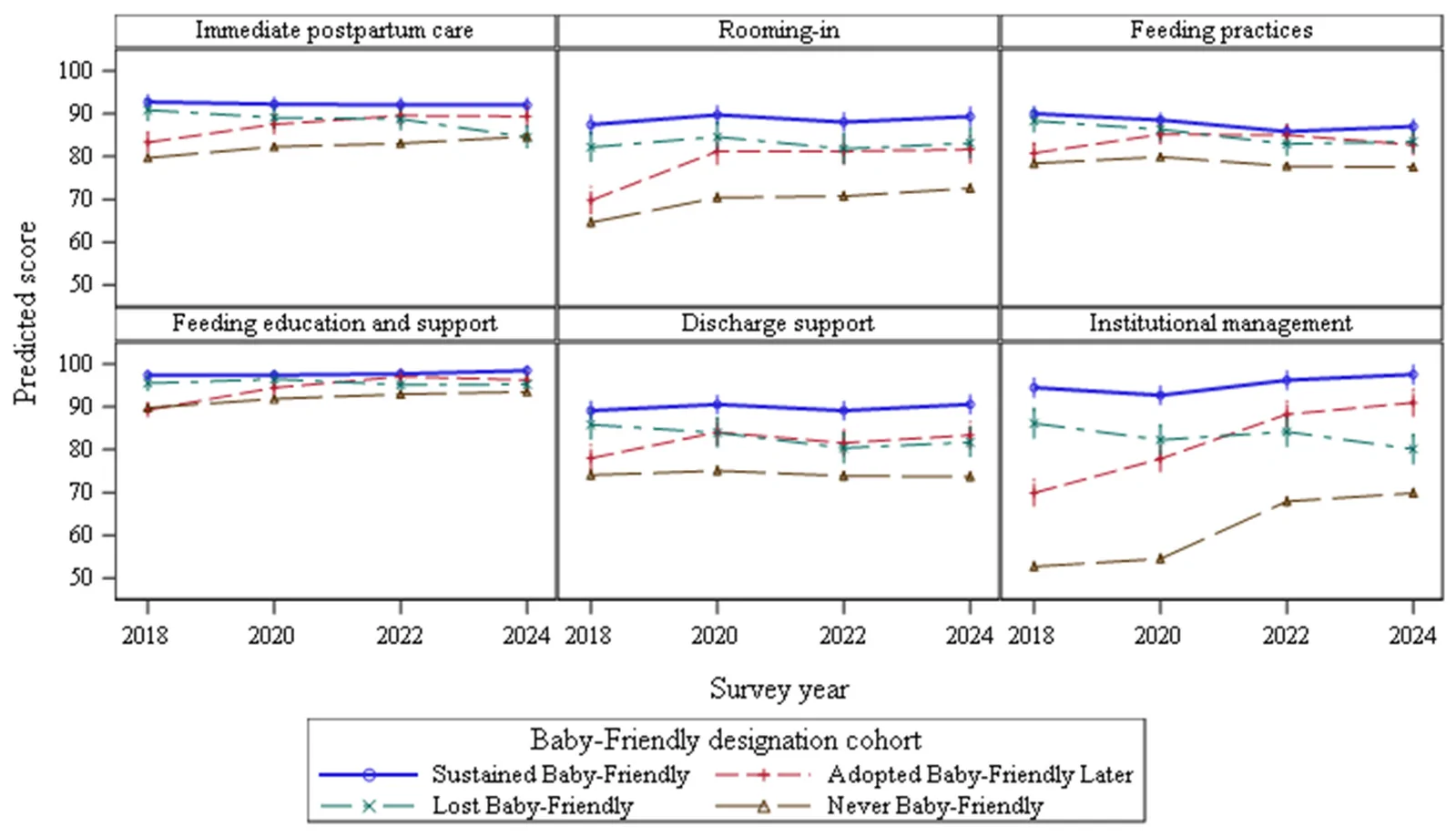
Domain-specific mPINC scores by Baby-Friendly designation cohort, 2018–2024.

**Table 1:** Descriptive Characteristics of Maternity Hospitals by Baby-Friendly Designation Cohort.

Characteristics at Most Recent Survey	Sustained Designation (N = 400)	Adopted Late (N = 190)	Lost Designation (N = 160)	Never Designated (N = 1554)	Total (N = 2304)
Hospital Ownership Type					
Government or Military	79 (19.8)	31 (16.3)	15 (9.4)	158 (10.2)	283
Non-profit, private	286 (71.5)	142 (74.7)	132 (82.5)	1131 (72.8)	1691
For-profit, private	35 (8.8)	17 (8.9)	13 (8.1)	265 (17.1)	330
Teaching Hospital					
Yes	326 (81.5)	146 (76.8)	126 (78.8)	1197 (77.0)	1795
No	74 (18.5)	44 (23.2)	34 (21.3)	357 (23.0)	509
Highest Level of Neonatal Care					
Level I: Well newborn nursery	108 (27.0)	90 (47.4)	54 (33.8)	669 (43.1)	921
Level II: Special care nursery	114 (28.5)	56 (29.5)	35 (21.9)	476 (30.6)	681
Level III: NICU	140 (35.0)	37 (19.5)	62 (38.8)	364 (23.4)	603
Level IV: Regional NICU	38 (9.5)	7 (3.7)	9 (5.6)	45 (2.9)	99
Region					
Western	75 (18.8)	26 (13.7)	32 (20.0)	187 (12.0)	320
Southwest	48 (12.0)	27 (14.2)	38 (23.8)	256 (16.5)	369
Southeast	70 (17.5)	43 (22.6)	21 (13.1)	261 (16.8)	395
Northeast	61 (15.3)	20 (10.5)	9 (5.6)	121 (7.8)	211
Mountain Plains	35 (8.8)	26 (13.7)	15 (9.4)	172 (11.1)	248
Midwest	66 (16.5)	31 (16.3)	37 (23.1)	393 (25.3)	527
Mid-Atlantic	45 (11.3)	17 (8.9)	8 (5.0)	164 (10.6)	234
Hospital Size (annual live births)					
<250	37 (9.3)	48 (25.3)	26 (16.3)	324 (20.8)	435
250–499	44 (11.0)	36 (18.9)	18 (11.3)	305 (19.6)	403
500–999	87 (21.8)	45 (23.7)	26 (16.3)	349 (22.5)	507
1000–1999	98 (24.5)	33 (17.4)	40 (25.0)	295 (19.0)	466
≥2000	134 (33.5)	28 (14.7)	50 (31.3)	281 (18.1)	493

**Table 2:** Predicted total and domain-specific mPINC scores by Baby-Friendly designation cohort, 2018–2024.

Outcome	Cohort	2018	2020	2022	2024	2024–2018
		Mean (95% CI)				Change (95% CI)
Total mPINC score	Sustained	91.8 (90.6, 93.0)	91.8 (90.6, 93.0)	91.5 (90.3, 92.7)	92.5 (91.3, 93.7)	0.67 (−0.71, 2.05)
	Adopted Later	78.4 (76.7, 80.1)	85.0 (83.3, 86.7)	87.1 (85.4, 88.8)	87.3 (85.6, 89.1)	8.94 (6.98, 10.90)
	Lost	88.1 (86.2, 90.0)	87.0 (85.1, 88.9)	85.5 (83.6, 87.4)	84.7 (82.8, 86.6)	−3.42 (−5.55, −1.28)
	Never	73.1 (72.5, 73.8)	75.6 (75.0, 76.2)	77.6 (77.0, 78.3)	78.6 (78.0, 79.2)	5.49 (4.77, 6.22)
Immediate postpartum care	Sustained	92.7 (91.0, 94.5)	92.2 (90.6, 93.9)	92.1 (90.4, 93.7)	92.0 (90.3, 93.7)	−0.72 (−2.80, 1.36)
	Adopted Later	83.3 (80.8, 85.7)	87.5 (85.1, 89.9)	89.6 (87.2, 91.9)	89.4 (86.9, 91.8)	6.10 (3.14, 9.06)
	Lost	90.8 (88.2, 93.5)	89.1 (86.4, 91.8)	88.7 (86.0, 91.4)	84.5 (81.9, 87.2)	−6.32 (−9.53, −3.10)
	Never	79.7 (78.8, 80.6)	82.3 (81.4, 83.1)	83.1 (82.2, 83.9)	84.6 (83.8, 85.5)	4.96 (3.86, 6.05)
Rooming-in	Sustained	87.4 (85.1, 89.7)	89.7 (87.6, 91.9)	88.0 (85.9, 90.2)	89.3 (87.1, 91.6)	1.91 (−0.80, 4.62)
	Adopted Later	69.7 (66.5, 73.0)	81.2 (78.0, 84.4)	81.2 (78.0, 84.3)	81.6 (78.4, 84.9)	11.90 (8.04, 15.76)
	Lost	82.2 (78.6, 85.7)	84.6 (81.0, 88.2)	81.8 (78.2, 85.4)	83.1 (79.6, 86.7)	0.96 (−3.24, 5.15)
	Never	64.6(63.4, 65.8)	70.4 (69.2, 71.5)	70.7 (69.5, 71.9)	72.6 (71.4, 73.7)	7.99 (6.56, 9.42)
Feeding practices	Sustained	90.0 (88.3, 91.8)	88.5 (86.9, 90.2)	85.8 (84.1, 87.5)	87.0 (85.3, 88.7)	−3.00 (−4.98, −1.03)
	Adopted Later	80.8 (78.3, 83.3)	85.2 (82.7, 87.6)	85.0 (82.6, 87.5)	82.7 (80.2, 85.2)	1.94 (−0.88, 4.76)
	Lost	88.3 (85.6, 91.0)	86.3 (83.6, 89.1)	82.9 (80.2, 85.7)	83.5 (80.8, 86.2)	−4.85 (−7.91, −1.78)
	Never	78.4 (77.5, 79.3)	79.9 (79.0, 80.8)	77.7 (76.9, 78.6)	77.5 (76.6, 78.4)	−0.88 (−1.92, 0.16)
Feeding education and support	Sustained	97.4 (96.2, 98.6)	97.4 (96.3, 98.6)	97.7 (96.5, 98.9)	98.5 (97.3, 99.7)	1.05 (−0.52, 2.63)
	Adopted Later	89.3 (87.6, 91.0)	94.5 (92.8, 96.1)	97.0 (95.4, 98.7)	96.3 (94.5, 98.0)	6.99 (4.74, 9.24)
	Lost	95.5 (93.6, 97.4)	96.5 (94.6, 98.4)	95.2 (93.3, 97.1)	95.2 (93.4, 97.1)	−0.28 (−2.72, 2.16)
	Never	89.8 (89.1, 90.4)	91.9 (91.3, 92.5)	92.9 (92.3, 93.6)	93.5 (92.9, 94.1)	3.74 (2.91, 4.57)
Discharge support	Sustained	89.1 (86.8, 91.4)	90.6 (88.4, 92.7)	89.1 (86.9, 91.3)	90.6 (88.3, 92.8)	1.47 (−1.28, 4.22)
	Adopted Later	78.0 (74.8, 81.2)	84.1 (80.9, 87.2)	81.6 (78.4, 84.7)	83.4 (80.1, 86.6)	5.39 (1.48, 9.31)
	Lost	85.8 (82.3, 89.4)	83.9 (80.4, 87.5)	80.4 (76.8, 84.0)	81.7 (78.2, 85.2)	−4.11 (−8.37, 0.14)
	Never	74.0 (72.8, 75.2)	75.1 (73.9, 76.2)	73.8 (72.7, 75.0)	73.7 (72.5, 74.9)	−0.33 (−1.78, 1.12)
Institutional management	Sustained	94.5 (92.2, 96.8)	92.7 (90.5, 94.9)	96.2 (94.0, 98.4)	97.6 (95.3, 99.8)	3.05 (0.41, 5.69)
	Adopted Later	69.9 (66.6, 73.1)	77.8 (74.7, 81.0)	88.3 (85.1, 91.4)	90.9 (87.7, 94.2)	21.07 (17.31, 24.83)
	Lost	86.1 (82.6, 89.7)	82.3 (78.7, 85.9)	84.2 (80.6, 87.8)	80.1 (76.6, 83.7)	−6.02 (−10.10, −1.93)
	Never	52.7 (51.5, 53.9)	54.5 (53.3, 55.6)	67.9 (66.7, 69.1)	69.8 (68.7, 71.0)	17.17 (15.78, 18.56)

## Data Availability

Upon request, data with hospital identifiers (i.e., hospital name and address) may be shared under a data use agreement between the Centers for Disease Control and Prevention and applicable state, tribal, local, and territorial health departments for the development of public health programs. Data without hospital identifiers may be released under data use agreements for additional approved purposes such as answering research questions. Email mpinc@cdc.gov to learn more and to receive a data request form.

## Abbreviations

The following abbreviations are used in this manuscript:

ACOG	American College of Obstetricians and Gynecologists
AIC	Akaike Information Criterion
APHA	American Public Health Association
BFHI	Baby-Friendly Hospital Initiative
CDC	Centers for Disease Control and Prevention
CI	Confidence interval
IRB	Institutional Review Board
mPINC	Maternity Practices in Infant Nutrition and Care
NICU	Neonatal intensive care unit
US	United States
WIC	Special Supplemental Nutrition Program for Women, Infants, and Children
